# Phospholipids at the Interface: Current Trends and Challenges

**DOI:** 10.3390/ijms140611767

**Published:** 2013-06-03

**Authors:** Roman Pichot, Richard L. Watson, Ian T. Norton

**Affiliations:** Centre for Formulation Engineering, School of Chemical Engineering, University of Birmingham, Birmingham, Edgbaston B15 2TT, UK; E-Mails: r.l.watson@bham.ac.uk (R.L.W.); nortonit@bham.ac.uk (I.T.N.)

**Keywords:** phospholipids, lecithin, air/water interface, oil/water interface, emulsions, foam, monolayer, ITIES

## Abstract

Phospholipids are one of the major structural elements of biological membranes. Due to their amphiphilic character, they can adopt various molecular assemblies when dispersed in water, such as bilayer vesicles or micelles, which give them unique interfacial properties and render them very attractive in terms of foam or emulsion stabilization. This article aims at reviewing the properties of phospholipids at the air/water and oil/water interfaces, as well as the recent advances in using these natural components as stabilizers, alone or in combination with other compounds such as proteins. A discussion regarding the challenges and opportunities offered by phospholipids-stabilized structure concludes the review.

## 1. Introduction

Phospholipids (PL) play a major role in very important functions of living organisms; they are the major component of biological membranes, are part of all major tissues and are concentrated in vital organs that require neuronal interactions. It is then not surprising that phospholipids properties, such as chemical or crystal structure, self-assembly, hydration, phase transition, *etc.* have been long studied and are nowadays well understood [1–3].

Phospholipids are a class of lipids that are formed of a phosphate-containing polar head-group attached to non-polar hydrocarbon chains. The nature of the head-group can be very diverse, with different functional groups attached to the phosphate groups, amongst which the most common are phosphatidic acid (PA), phosphatidylcholine (PC), phosphatidylethanolamine (PE) or phosphatidylserine (PS) [2]. The type of fatty acid chains also varies and depends in both the chain length and the carbon saturation. Lecithin is likely the most common form of phospholipids, but the term “lecithin” can be rather confusing. When used in biochemistry, lecithin means PC, but when used in food, lecithin is a mixture of various PLs amongst which PC is the major component. On the other hand, PC is the name of a class of phospholipids which all have the same polar head-group but various fatty acid chains. Because lecithin is mainly formed of PC, it is common use to name PC as lecithin or lecithin as PC. In this article, the terms PC and lecithin will be used as mentioned by cited articles’ authors.

Phospholipids are amphiphilic compounds; the phosphate polar head-group composes the hydrophilic moiety, and the backbone, as well as the fatty acids, the hydrophobic moiety. The solubility of phospholipids in water depends on both the head-group polar head type and the hydrocarbon chain length [4,5]. Four classes of phospholipids can be distinguished as a function of PL solubility: class I includes insoluble PLs, that do not absorb water at all (e.g., waxes); class II, PLs with very low solubility, which swell in water (e.g., long-chain phosphatidylcholine, phosphatidylethanolamine or sphingomyelin); class IIIA, soluble PLs forming lyotropic liquid crystals at low water content (e.g., lysolecithins); class IIIB, relatively rare, soluble PLs forming micelles above the critical micelle concentration (cmc), but no crystalline structure (e.g., saponins). Because of their amphiphilic character, phospholipids exhibit various thermotropic and lyotropic phase structures, from solid-like lamellar to liquid phases. Most of the PLs exhibit a 3-D lamellar crystalline structure at low temperature and/or hydration level. Other solid-like structures such as 2-D lamellar crystals or different gel phases can be formed by PLs [6]. Phase transition, which can be classified as solid-solid, chain melting or fluid phase transitions, is mainly induced by temperature variation; by increasing the temperature above a certain point, hydrocarbon chains become liquid which induces a transition from a solid-like to a liquid-like structure. This critical chain-melting temperature depends on the type and the length of the PL hydrocarbon chain; phase transitions are shifted towards higher temperatures when PL chain length is increased [7]. Phospholipids, within their liquid structure, tend to form bilayer structures when swollen in water; when swollen in oil, the bilayer structure tends to separate into two monolayers.

## 2. Interfacial Properties of Phospholipids

### 2.1. Properties of Phospholipids at the Air/Water Interface

**Figure 1:**
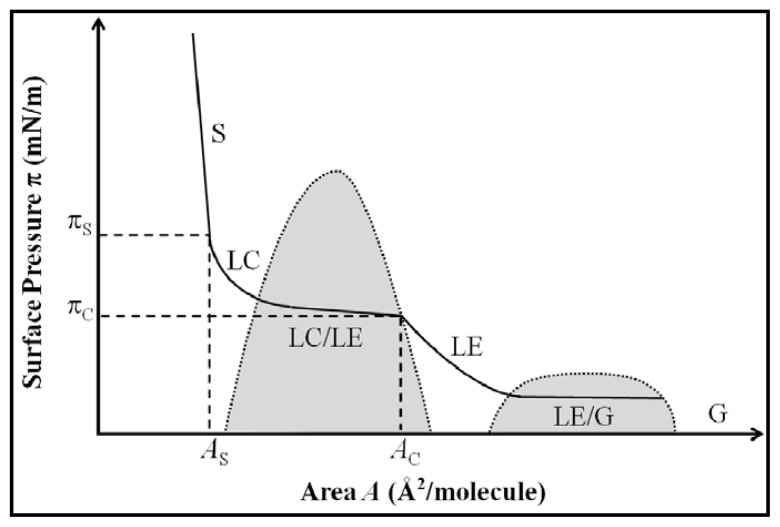
Representation of phospholipid surface pressure (π) *vs.* area per molecule (A) isotherm. Grey areas represent the coexistence regions (LC-LE and LE-G). S: solid-like structure region; LC: liquid-condensed region; LE: liquid-expanded region; G: gaseous region. π_S_ and *A*_S_ are the transition pressure and area, respectively, between liquid-like and solid-like structures. π_C_ and *A*_C_ are the critical pressure and area, respectively, between LE and LC/LE coexistence regions (Adapted from Möhwald [11]).

Because of their critical role in structuring and stabilizing biological interfaces such as cell membranes, the interactions between PLs and water have received a lot of interest, in particular their influence on the air/water (a/w) interface. More generally, a lot of studies have been carried out on the effect of amphiphilic molecules, such as protein, low molecular weight surfactants or polymers, on the interface. Recent reviews summarize the main findings and conclusions [8,9]. Many techniques have been developed to characterize interfaces, including thermodynamic measurements, optical techniques (fluorescence microscopy or spectroscopy), neutron scattering, infrared techniques, *etc.* and have been reviewed in detail by Möhwald [10]. Nonetheless, amongst these techniques, the measurement of lateral surface pressure (π) as a function of the molecular area (A) is the most commonly used technique to characterize the behavior of phospholipids at the air/water—and also oil/water (o/w)—interface, due to ease of implementation. The surface pressure is defined as the difference in surface tension measured between an uncontaminated surface (γ_0_) and a surface active agent-covered surface (γ), *i.e.*, π = γ_0_ − γ. A hypothetical (π, A) isotherm is given in Figure 1.

One of the first attempts to characterize PL behaviour at the air/water interface was made by Phillips and Hauser, who investigated the spreading of various phosphatidylcholines and phosphatidylethanolamine at such an interface [12]. By measuring the equilibrium spreading pressure π_e_, pressure at which the monolayer (or bilayer) is in equilibrium with the bulk phase, as a function of temperature, the authors showed these two PLs have antagonistic behaviour; PE spreads at the interface from crystalline structure (*i.e.*, at low temperature) to liquid-like structure (at high temperature), while PCs can only start to spread when chain melting occurs (solid-like to liquid-like phase transition). This difference was attributed to (i) the low van der Waals interactions between the PE hydrocarbon chains, which allow the crystals to spread, and (ii) the necessary hydration of the PC crystals to adsorb at the interface, which corresponds to liquid-like phase transition. The authors also demonstrated that anhydrous PC crystals do not affect the surface tensions.

A few years later, Albrecht *et al.* published two articles in which they investigated the influence of temperature and surface pressure on the properties of many phospholipids, such as DPPC, DHPC, DMPA, α-DPL, α-DML or β-DPL, at the air/water interface [13,14]. The authors noticed a change in the (π, A) curve of compressed DMPA monolayer as temperature increases; a plateau appeared above a critical temperature and upon a critical compression (π_c_, *A*_C_), which itself depends on the temperature. Further compression of the interface leads to an abrupt increase of the lateral pressure (π_s_, *As*). This behaviour of DMPA at the air/water interface was attributed to phase changes. When an expanded film is compressed, a liquid-expanded phase (LE) is produced. Further compression (at pressure above π_c_) leads to a more condensed phase (liquid-condensed phase—LC). At high compression (at pressure above π_s_), a phase designated as solid is formed. This LE to LC phase transition was investigated by many others in order to determine the order of transition [15,16]. Because the plateau of the curve (π, A) is not perfectly horizontal, the first order transition has been questioned. Miller and Möhwald [15] and Pallas and Pethica [16] have shown that the non-horizontal character of the curve is not due to a second order transition but mainly to impurities adsorbed at the interface.

Spreading of phospholipids at the air/water interface was investigated in detail during the next decades, and the main conclusions were reported in a few reviews at the beginning of the 1990s [11,17]. The interest in phase transitions during monolayer compression experiments remained unchanged and other measurement techniques allow both confirmation of the conclusions aforementioned and extension of the knowledge of phospholipid behaviour at the air/water interface. For example, molecular orientation and structural changes at the interface were studied by surface potential measurements [10,18] and infrared spectroscopy [18–20] techniques. Using the former technique, it was shown that, within the LE phase, monolayer changes due to compression are only due to molecular density, not to molecular arrangement. The first hint of the coexistence of different phases at a given compression was also obtained using surface potential measurements.

This was confirmed by optical microscopy [21,22]. Fluorescence microscopy also allows the visualisation of PL domains at the air/water interface [23,24]. Flörsheimer and Möhwald [23] studied the fusion of small DMPE domains, formed using an electric field, at the interface. Van der Waals forces and electrostatic repulsion acting between liquid crystalline phase areas are responsible for either fusion or separation of the PL domains. For domains to link, van der Waals forces must overcome electrostatic repulsion; domain fusion can only be achieved if PL orientation at the interface favours close hydrocarbon chain interactions and prevents head-group dipole repulsion. The fusion of PL domains at the interface was shown to be irreversible; linked domains did not split under further expansion of the interface.

The dynamic properties of phospholipids at the air/water interface have been studied in detail by Makino and Yoshikawa [25]. The authors investigated the response of an unsaturated phospholipid (DOPC) film to periodic changes in the surface area. A DOPC film under compression and expansion behaved as a non-linear viscoelastic material; the curve (π, A) did not form a genuine elliptic loop as expected for a linear viscoelastic film, but a ‘croissant’ shape. The authors also noticed that the ellipse deformation was more pronounced as the amplitude of the period change was increased. This study also emphasized the structure of DOPC at the interface under dynamic conditions. Two different PL structures at the interface were suggested, as a function of the compression/expansion rate. At slow film compression, PL domains are formed at the interface by fusion of PL molecules, which gradually increases the surface pressure. This observation is consistent with the aforementioned conclusion of Flörsheimer and Möhwald [23]. However, under fast compression, the authors argued that there is not enough time for the domains to link with each other and grow at the interface. This results in collision of domains at high compression, which then form large PL aggregates presenting an oil droplet-like structure.

**Figure 2:**
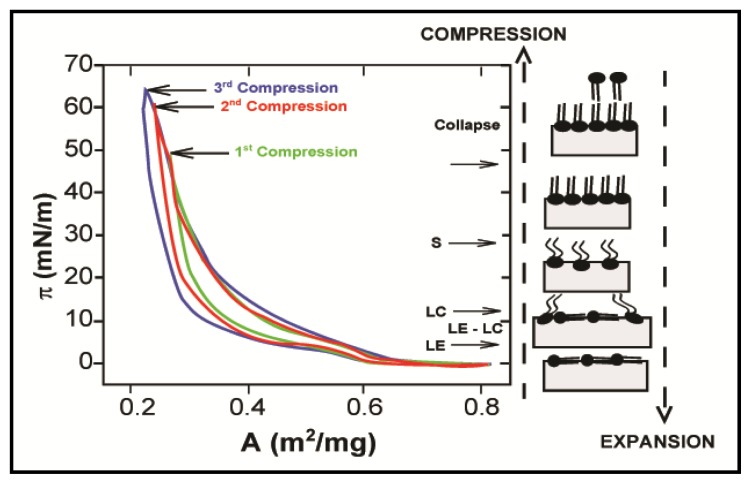
Hysteresis in π–A isotherms for DPPC monolayers at the air–water interface after continuous compression–expansion cycles at pH 7. The arrows indicate the equilibrium spreading pressure (π_e_) and the transitions that take place in the monolayer during a compression–expansion cycle between LE, LC and solid phases. Reproduced with permission from Rodríguez Niño *et al.* [26], Copyright (2008) Elsevier.

Such non-linear viscoelastic behaviour of PLs at the a/w interface was also reported by Rodríguez Niño *et al.* who investigated relaxation phenomena in both DOPC and DPPC monolayers at the a/w interface [26]. Hysteresis in the (π, A) curve during a compression-expansion cycle for both phospholipids was observed (Figure 2). However, differences were noticed between the two PLs; DPPC isotherm hysteresis occurs regardless of the compression level. Moreover, (π, A) isotherms for consecutive compression-expansion cycles were shifted towards the lower molecular area. This displacement shows an irreversible monolayer loss. Hysteresis in the DOPC isotherm is only observed above a critical compression. Below this surface pressure, DOPC isotherms do not show hysteresis and are reproducible. The authors concluded that relaxation phenomena were driven by different mechanisms depending on whether the surface pressure is lower or higher than the equilibrium spreading pressure (π_e_); below π_e_, relaxation phenomena are controlled by molecular re-arrangement (e.g., self-assembly), while at higher pressure they are mainly due to molecular interactions, such as nucleation, or to opposite mechanisms, such as monolayer collapse or desorption, that occur together at the same time.

Spreading of phospholipid bilayers, which are a major structural element of biological membranes, at the air/water interface has also received a lot of interest, in particular the transitions of PLs between monolayers and bilayers [27–30]. The main findings of these studies were summarised by Mansour and Zografi, who also investigated the relationships between equilibrium spreading pressure and phase equilibria of bilayers and monolayers at the interface for three different PLs (PC, PG and PE) [31]. The authors measured and compared equilibrium spreading pressures π_e_ of PL bilayers and critical spreading pressure π_c_ (pressure at which the transition between monolayer coexistence LE–LC region and LE state occurs) of monolayers, for various temperatures. Above the bilayer gel-to-liquid crystalline phase transition temperature *T*_m_, bilayers of PC, PG or PE, all within the liquid-crystalline state, spread to form LE monolayers. Reciprocally, above the critical spreading temperature *T*_c_, LE monolayers of the three PLs above *T*_m_ collapse to form thermodynamically stable liquid-crystalline PL bilayers, π_e_ and π_c_ being very close ~45 mN/m. At temperatures below *T*_m_, the authors showed that PE behaved in a different way to PC and PG. Bilayers of PC and PG exhibit a very low π_e_ (nearly 0 mN/m) and spread to form a gaseous monolayer, while LC monolayers (below *T*_c_) collapse at ~45 mN/m to form metastable liquid crystalline bilayers. Unlike PC and PG, gel and crystal PE bilayers can be formed if the temperature is above or below the crystal-to-gel phase transition temperature *T*_s_, respectively. Gel bilayers would spread to LC monolayer while crystal bilayer would spread to gaseous monolayers. LC monolayers collapse to form gel bilayers, regardless of the temperature. These differences between PC/PG and PE behavior at the a/w interface were attributed to PE polar headgroup—headgroup hydrogen-bonding and electrostatic interactions in both bilayers and monolayers. PE phospholipids are able to pack more tightly in the bilayer and monolayer states than PC and PG phospholipids. Despite the observations and hypothesis made in this study, the reasons of such different behaviour are not yet totally understood.

In spite of the fact that most of the studies about PL at the a/w interface were carried out to characterize spreading of phospholipids at the interface, other phospholipid surface properties such as surface viscosity [32–35] or surface tension [36–39] have also been investigated. It was shown by Hayashi and co-workers [33,40,41] that the surface viscosity of PC was dependent on the monolayer state; low viscosities were observed under LE monolayer state, while high viscosities, similar to those of polymer films at the air/water interface, were found under LC monolayer state. High surface viscosities were also observed by Evans, who measured surface viscosities of various phospholipids (PC, PE and PG) in order to determine the presence of aggregates at the interface [32]. The author also showed nonetheless that PL surface viscosity was dependent on the properties of the hydrocarbon chain. For instance, viscosity of *trans*- or *cis*-unsaturated PC could not be detected (due to very low viscosity values) while saturated PC at the interface exhibited very high viscosity. Evans also concluded that the addition of small amounts of steroid such as cholesterol resulted in a large reduction of surface viscosity. This change in surface viscosity by addition of cholesterol was confirmed by Joos [42] and Vrânceanu *et al.*, who measured surface rheology (elasticity and viscosity) of PC/cholesterol mixtures [35]. Surface rheology was first measured by Krägel *et al.*, who investigated the shear and dilational rheological behaviour of DPPC and DMPE at the air/water interface [34]. Shear rheology showed that surface viscosity of DMPE and DPPC increases from very low to very high values after the transition from LE to LC monolayer state. The dilational elasticity and viscosity both exhibit a strong dependence in surface pressure with a minimum in the LE–LC coexistence region. In the LC region, both viscosity and elasticity increase. Such dependence is explained by considering changes in area both within and out of the coexistence region. Within the coexistence region, changes in area do not result in changes in surface pressure. Thus, viscosity and elasticity are both very low. On the contrary, below or above the LE–LC region, area changes result in surface pressure changes. Consequently, changes in the dilational elasticity and viscosity are observed. The strong elastic character of phospholipids, in particular lecithin, was also reported by Norton and co-workers [43,44]. Figaszewski and co-workers, through a series of publications [37,39,45–47], have attempted to characterize surface behavior of phospholipids by measuring surface tension under various conditions of pH, PL mixtures and mixtures of PL and steroids such as cholesterol. In many of these studies, the authors have developed theoretical models, in order to predict the effect of pH on the surface tension. These models were compared to experimental data. Interfacial tension exhibited an optimum around PL isoelectric point, *i.e.*, pH ~4.5. However, it seems that the nature of this optimum depends on layer state (monolayer or bilayer) and the type of phospholipids. For example, surface tension of systems containing phosphatidylcholine only has a maximum around pH ~4.5, while a minimum is observed when mixed with PS to form a bilayer interface. The latter system also exhibited a much lower surface tension (10-fold).

### 2.2. Properties of Phospholipids at the Oil/Water Interface

Characterization of soluble or insoluble molecule adsorption at liquid/liquid interfaces, e.g., oil/water, is more complex and difficult to assess experimentally than that of air/liquid interfaces. Even though first studies on monolayer isotherms at oil/water interfaces were published in the 1960s [48] and despite significant progress in measurement techniques for the last 50 years, the number of studies released on the properties of phospholipids at the oil/water interface remains limited, compared to the extended literature on PLs at the a/w interface.

Adsorption of PLs at the interface between two immiscible electrolyte solutions (ITIES) was first studied in the 1980s by Girault and Schiffrin [49,50], shortly followed by Wandlowski *et al.* [51,52]. ITIES properties allow the control of the potential difference between the two liquids, providing the possibility of studying the stability and ion permeability of the phospholipid monolayer as a function of potential difference. These early works showed that PLs’ monolayer stability at polar liquid-liquid interfaces strongly depends on the interfacial inner potential difference and the pH at the interface; a stable monolayer is formed from zwitterionic phospholipids (obtained under negative potentials) but breaks down when potentials increase transforms PLs into ionic species. Further investigations took place within the following two decades. Ion transfer through the PL monolayer was studied in details by Kontturi and co-workers [53–55]; the role of aqueous cations on the stability of the PL monolayer was investigated by Kakiuchi *et al.* [56,57]; and work on the dynamics of PL monolayer at ITIES was undertaken by Samec *et al.* [58]. For more information on the behavior of phospholipids at ITIES, one can be referred to the recent review article by Santos *et al.* [59].

In fact, the first extended investigation was published in the early 1990s by Shchipunov and Kolpakov [60]. The authors questioned the assumption made in a few previous studies [61–63] which stated that monomolecular films of PL were formed at the oil/water (o/w) interface, similarly to the air/water interface; as many PLs are soluble (or at least partially soluble) in oil, the adsorption and organization of PLs at the interface may significantly differ from air/water to oil/water interfaces.

Shchipunov and Kolpakov first studied the adsorption of PC at the alkane (n-heptane or n-decane)/water interface. Interfacial tension measurement at various PC concentrations allowed the authors to determine adsorption kinetics; at early stages, adsorption is limited by the diffusion of PL through the bulk phase to reach the interfacial boundary, while at a later period, the penetration of PL molecules into the PL adsorption layer, as well as the possible re-orientation of PL molecules within this layer, is the limiting factor. The authors also observed that thermodynamic equilibrium is obtained after a very long period of time, ~12–15 h. Further analysis also showed a discrepancy between adsorption data and data obtained for the theoretical monolayer model, which could be overcome by using a multilayer model. The existence of a three-dimensional interfacial structure was demonstrated as follows. When entering the interfacial boundary, interactions between water and PC molecules result in PL hydration and a sharp drop in solubility. These hydrated PCs tend to bind with each other, which prevents their desorption from the interface into the bulk oil phase. Having shown that the PC adsorption layer must be at least three molecules thick, the authors argued that the formation of the 3-dimensional adsorption layer starts when the number of molecules adsorbed and bound at the interface exceeds the number of molecules necessary for interfacial monomolecular coverage. Based on the work by Onigo and Onishi [64] and Friberg *et al.* [65] on the formation of lecithin liquid-crystalline state at the oil/water interface, Shchipunov and Kolpakov concluded that hydrated PC could potentially form liquid-crystalline states. Within the interfacial boundary and for a given system (depending upon PL concentration and type, pH, type of oil), phospholipids can exist in a variety of structural forms.

The multilayer model aforementioned is highly dependent on the type of phospholipids and seems restricted to class II and eventually class IIIA PLs. As class I PLs do not adsorb water molecules, binding at the interface would be very limited; as class IIIB PLs are soluble and form micelles in water, one could think that they would behave as classical surfactants such as polysorbates for example. The effect of PL solubility on o/w interfacial structure was studied by Li *et al.* [66], using axisymmetric drop shape analysis. Nonetheless, PLs were chosen according to their solubility in the oil phase; DPPC and DMPE are soluble in chloroform but insoluble in dodecane. Structural differences at the interface were noticed by the authors. When insoluble in oil, DMPE and DPPC exhibited similar characteristic behaviour of PLs at the air/water interface; analysis of the (π, A) isotherm showed phase transitions from gas/liquid states at low compression to more condensed states at high compression, with aliphatic chains forming a liquid crystal layer. The authors stated that insoluble monolayers are formed by mechanical actions (compression or expansion). Soluble phospholipids adsorb at the interface in a different manner. An adsorption layer is formed by penetration and orientation of PL molecules into the interfacial boundary, which renders the interfacial structure strongly dependent on the bulk phase. No evidence of a coexistence region was observed. Nonetheless, it was proved that above the critical aggregation concentration (CAC), a monomolecular layer in the liquid-crystalline state was formed at the chloroform/water interface. This was explained by dehydration of headgroups due to the aggregation of PLs, which confirms observations by Shchipunov and Kolpakov [60].

A direct comparison of PL at the air/water and oil/water interface was published by Grandell and Murtomäki [67]. Surface pressures obtained at the air/water interface for both DSPC and DPPC were much higher than at the 1,2-dichloroethane/water interface. The authors stated this was due to the penetration of oil molecules present into the interfacial boundary within the PL monolayer, which results in screening van der Waals interactions between hydrocarbon chains. This creates disorder within the monolayer, which leads to a more expanded liquid-crystalline phase at the oil/water interface. Evidence was also given that proves the structural transition from bilayer to multilayer in the presence of DSPC as the pressure increases. No plateau region or phase transition was observed, which was explained by the small size of oil molecules that can move freely within the interfacial layer. However, the absence of a phase transition was also observed by Li *et al.* in the case of oil soluble phospholipids [66]. Consequently, one can wonder if this behavior in the study by Grandell and Murtomäki could not be induced by the solubility of DSPC and DPPC in 1,2-dichloroethane, which is not discussed by the authors.

**Figure 3:**
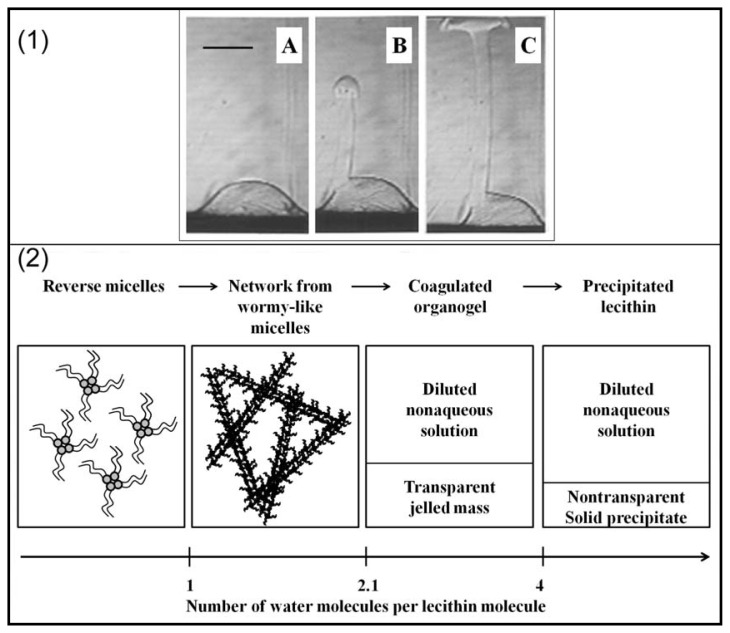
(**1**) Photographs showing a minor part of an aqueous solution at the bottom and a nonaqueous solution above. (**A**–**C**) Formation of an organogel diffusing from the interface through the oil phase; photographs were taken after 3.0, 3.5, and 3.8 min, respectively (the bar indicates 1 mm); (**2**) Schematic representation of the phase and pseudophase transitions in a 1% *w*/*v* lecithin solution after addition of water. Adapted with permission from Shchipunov and Schmiedel [68], Copyright (1996) American Chemical Society.

**Figure 4:**
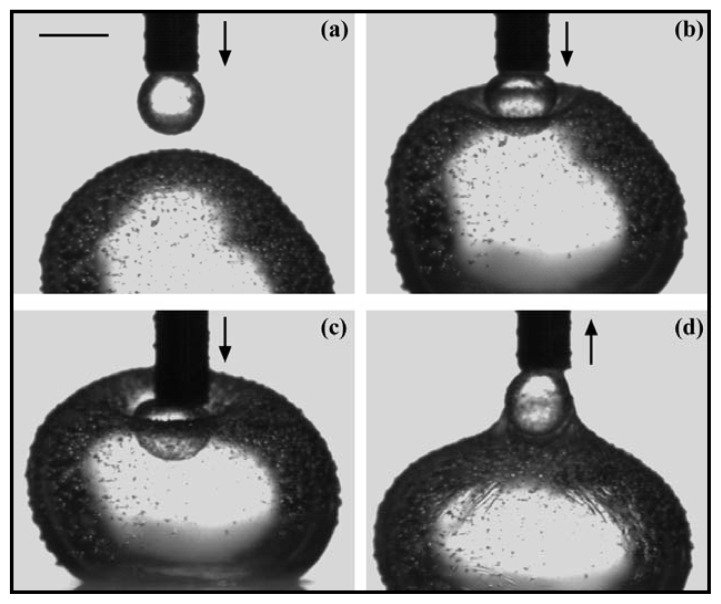
Visualization of the visco-elastic properties of lecithin at the oil/water interface. The system includes a silica particles-in-water dispersion (1%) and a solution of lecithin in sunflower oil (0.1%). From pictures (**a**) to (**c**), the needle is pushing down into the water droplet; on picture (**d**) the needle is pulling up out of the water droplet (the bar represents 2 mm). Reprinted with permission from Pichot *et al.* [44], Copyright (2012) Elsevier.

Most of the investigations about PLs at the o/w interface were carried out with PC as phospholipid. Shchipunov and Schmiedel dedicated part of their work to the phase behaviour of soybean lecithin at the interface [68]; the authors focused on the phase formation and transition taking place at the alkane/water interface. Formation and alteration of the PL layer at the interface was visualized by polarized light microscopy (Figure 3). The formation of a liquid phase flowing from the interface upper layer through the oil phase was observed and explained as follows. Hydrated lecithin molecules adsorbed at the interface form aggregates (PC concentration above CAC) of which the structure changes from micelles to a PL micelle network. This change is followed by the formation of a gel-like structure of PLs in the oil phase in contact with the interface. This allows the penetration of more water molecules in the gel-like structure resulting in the formation of an organogel which detaches from the interface because of the density difference. The authors also confirmed in this study the conclusions made by Ma *et al.* [69], *i.e.*, that the formation of precipitates at the interface tend to slow down adsorption processes or the formation of a PL layer at the interface. The strong elastic behavior of lecithin at the air/water interface mentioned in Section 2.2 was also observed at the oil/water interface by Pichot *et al.* [44]. As can be seen in Figure 4, adsorption of lecithin at the interface prevented two water droplets merging. Figure 4b,c shows the deformation of the bottom droplet under compression of the droplet attached to the needle. Both droplets having the same composition, this clearly demonstrates that adsorption of lecithin around the water droplet confers to the interface a very strong elastic character. In a similar manner, demonstration is made of the viscous character of the interface in the presence of lecithin by analyzing Figure 4d. When the top droplet is moved away from the bottom droplet, the latter clearly deforms, forming a layer around the small droplet. This was assumed to be due to the viscous behavior of lecithin at the interface.

As aforementioned, phospholipids diffuse through the bulk phase to reach the interface and are capable of forming various types of interfacial structures. Nonetheless, the dynamics of PL molecules at the oil/water interface remains poorly understood due to lack of studies. Adalsteinsson and Yu investigated the DLPC lateral diffusion at the a/w and heptane/water interface using fluorescence recovery after photobleaching (FRAP) technique [70]. Diffusion coefficients of PL at both interfaces were deduced from (π, A) isotherm measurement and plotted versus the molecular area. The authors showed that at high surface density, diffusion coefficients of PL at the a/w and o/w interfaces were very similar, while at low surface density, diffusion at the a/w interface was faster than at the o/w interface. It was argued that at low surface density, the diffusion of PL was controlled by the viscosity of the nonaqueous phase; oil viscosity being much higher than the water one, diffusion at the oil water interface was slower. By increasing the surface density, PL monolayer is formed at the interface and the diffusion is then controlled by the viscosity of the monolayer, becoming independent of the nature of the nonaqueous phase. The dependence of PL diffusion through the interface on the surrounding environment was also demonstrated by Negishi *et al.* who showed that the PE diffusion at a mineral oil/water interface decreases with the oil and water viscosity (η) as: D~(η_water_ + η_oil_)^−0.85^[71]. This work was continued by Walder *et al.* who studied the effect of oil viscosity on the diffusion of PE at the o/w interface, also using FRAP technique [72]. The authors also observed that the diffusion coefficient decreased by increasing the concentration of PL at the interface, reaching a plateau at high concentration, at which PL domains of condensed phase were observed. In the presence of low viscosity oils (<1500 cP), diffusion of both single PL molecules and domains follows the Stokes-Einstein model; PE movement within the interface is dictated by the oil viscosity. At higher oil viscosity, domain diffusion was too slow to be measured. PE molecules exhibit surprisingly high diffusion coefficients with viscous oils. As no conclusion could be made to explain this behavior, two diffusion modes were suggested. In the first mode, called “hopping” mode, highly viscous oil is assimilated to a solid surface, from which PL adsorbed molecules become partially detached. This involves complicated transition states at the interface. The second mode involves desorption-mediated interfacial diffusion; PL molecules desorb completely from the interface through the aqueous phase. Because of higher affinity for oil, diffusion through the water phase is very limited and PL molecules re-adsorb at the interface.

Under certain bulk and temperature conditions, phospholipids can form vesicles, also called liposomes [73–75]. Despite their real potential for use in food or pharmaceutics, the study of PL vesicles at the oil/water interface has received only little interest so far. Yang *et al.* investigated the effect of various electrolytes and mixing ratio of PS and PC vesicles on their adsorption at the o/w interface [76]. The presence of salt was found to enhance the adsorption of vesicles at the interface, as van der Waals forces increase. Nonetheless, if DLVO theory allowed the authors to explain the role of NaCl and MgCl_2_ in the better adhesion of vesicles at the interface, the effect of LaCl_3_ could not be explained. Mixtures of PS and PC vesicles also result in better adhesion of vesicles at the interface compared to PC vesicles only. The authors suggested this was due to better packing of mixtures of vesicles at the interface. Nonetheless, microscopy observation and vesicle size determination seem to be necessary to confirm this hypothesis. Transport of PL vesicles through the o/w interface was studied by Hase *et al.* [77]. A micron-size vesicle was formed in a solution of DOPC in mineral oil by injecting a small drop of water through a glass capillary, which was spontaneously covered by a PL layer. The vesicle was then passed through the interface a few times by using micromanipulation techniques. Modifying the outer environment of the vesicle resulted in structural changes of the PL layer around the vesicle; crossing the interface to the water phase, the external structure of the vesicle was formed by an oil layer trapped between two PL layers. This structure was not stable with time, as internal water diffused through the double layer, which leads to the formation of a small oil droplet covered by phospholipid molecules. If the vesicle was carried through the interface to the oil phase before its collapse, a multilayer vesicle was formed and presented the following structure: water droplet→DOPC layer→oil layer→DOPC layer→water molecules→DOPC layer. Nonetheless, the presence of water molecules tends to destabilize the multilayer structure, which collapses to the initial water/DOPC vesicle.

The surface composition PC-water-triolein system has been determined by Mitsche *et al.*, who used comparison of compression isotherms obtained from both Langmuir trough and drop tensiometry [78]. Demonstration was first made that this method could be used for the calculation of the concentration of PC at the surface of an air bubble in water. Langmuir trough analysis allows the determination of the area per molecule at the collapse point. Knowing the surface area of the drop/bubble and the area per molecule, the surface concentration as a function of surface pressure can easily be calculated. The same methodology was used to determine the concentration of PC at the triolein drop surface. This relatively simple technique can be used to determine the surface concentration and coverage of oil emulsion droplets in the presence of PLs, parameters of great interest for emulsion formation and stability study but usually very difficult to calculate or estimate.

## 3. Applications

### 3.1. Emulsions

Due to their amphiphilic character, as well as their interfacial properties and their natural sources of production (egg yolk, soybean, *etc.*), the use of phospholipids as emulsifiers have been investigated since the late 1960s. A consequent number of papers have been published; most of them dealing with potential applications of PLs-stabilized emulsions for the pharmaceutical industry, a few of them for the food or cosmetic industry.

The first efforts in order to understand the stabilization mechanisms of emulsions by PLs are attributed to Friberg and co-workers who investigated the effect of liquid crystalline layers at the interface on the emulsion stability [79–81]. After a phase diagram for the system water–lecithin–trioctanoin was determined [80], it was shown that emulsion stability was enhanced by the presence of a PL liquid crystalline layer at the interface [81]. Nonetheless, this stability has to be understood as assessed in quiescent conditions, as under centrifugation the crystal layer can be removed from the interface. Long-term stability was also shown to depend on the dispersion method of the phospholipids; emulsions were more stable when aggregates of the liquid crystalline phases were visible. Friberg also shows that the ratio between the Hamaker constant for the liquid and the one for the emulsified droplets was more relevant to assess emulsion stability than the thickness of the adsorbed PL layer. In other words, increasing the repulsion between droplets or reducing droplet attraction is more important to preserve emulsion long-term stability than the presence of single or multilayer at the interface.

This work was pursued by many researchers. Phospholipid emulsifiers were proved to provide stability to emulsions by acting as both a mechanical and electrostatic barrier to coalescence [82]. Rydhag and Wilton investigated how the composition of PC affects the emulsion stability [83]. Only oil-in-water (o/w) emulsions were formed and their stability was enhanced by a multilayer of PL around the oil droplets; an interfacial film corresponding to at least two PL layers is necessary to guarantee emulsion stability. The authors also studied the swelling of the various PCs at the interface and showed that the swelling behavior was dependent on the concentration of other PLs (PE, PA and PI); swelling is enhanced by the presence of negatively charged PLs, such as PI and PA, as these PLs are more soluble in water and adsorbed more water molecules. This work also complemented the study by Friberg [81] by demonstrating that charged PLs increase droplet repulsion, which ensure better emulsion stability. Nonetheless, this conclusion was questioned by Washington *et al.* who studied the electrokinetic properties of emulsions stabilized by a mixture of PLs (PC, PG, PS and PA). The authors also observed that emulsion droplet flocculation is decreased or prevented by negatively charged phospholipids, but they argued that, in the presence of electrolytes such as calcium chloride, emulsions stabilized by both charged and uncharged PLs were more prone to destabilisation due to flocculation than those only stabilized by uncharged PLs. It is worth noting that none of these studies considered the interfacial behavior of PLs under dynamic conditions; as coalescence occurs because of emulsifier desorption or displacement along the interface (Marangoni effect), it could have been interesting to study the response of these PLs or mixtures of PLs/electrolytes adsorbed at the interface when this one is stretched or compressed.

Emulsion stability has also been assessed as a function of PL interfacial and bulk properties. The ability of PC, PE and PS to act as emulsifiers has been investigated by Handa *et al.* [84]. Emulsions were shown to be more stable when produced with PC as emulsifier (in comparison to PS or PE) because PC is able to form a more compact monolayer at the interface. The monolayer formed by PC is in a LC state while those formed by PS and PE are in a LE phase. It was argued that more molecules of oil are present within the interfacial boundary when PE and PS are used as emulsifiers which enhances droplet coalescence. Cornelus *et al.* studied the effect of PL structure in the bulk phase on the stability of highly concentrated o/w emulsions [85]. Lecithin was dispersed in fluorocarbon in various manners to obtain three different structures: PC aggregates as “pre-liposomes”, multilayer liposomes (MLV) and small unilamellar vesicles (SUV). Emulsions prepared with pre-liposomes dispersion were significantly smaller and more stable than the ones prepared with MLV or SUV dispersions. Even though the authors showed that the adsorption of PLs was promoted when dispersed as pre-liposomes, thus suggesting a thicker interfacial layer, the differences in emulsion stability were not explained and further investigations would be required.

**Figure 5:**
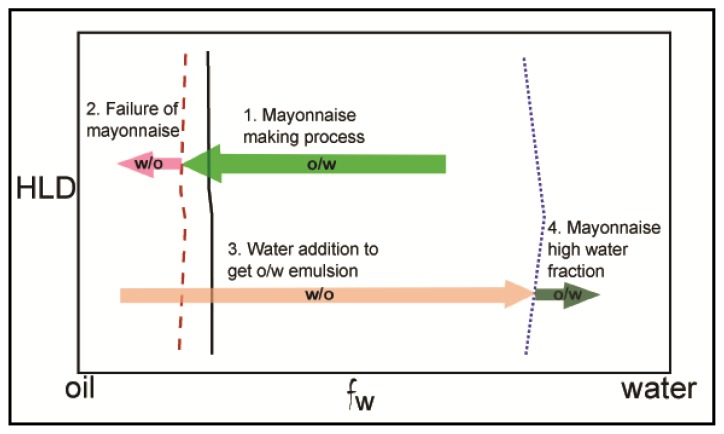
Schematic presentation of mayonnaise making process. Catastrophic inversion lines (—) at direct emulsification; and at dynamic emulsification (- - -) when oil is added and (…) when water is added. Reproduced with permission from Thakur *et al.* [86], Copyright (2008) Colloids and Surfaces A: Physicochemical and Engineering Aspects.

The effect of other parameters, such as pH or water/oil ratio, on the emulsion stability has been little investigated, except for very specific cases. Phospholipids easily undergo hydrolytic splitting of the ester bond in both acidic and alkaline media, limiting physical stability to pH 7 [87]. This is with regard to breaking up into fatty acid and other components with 1N acid or base. In a series of publications [86,88], Thakur *et al.* studied the effect of water volume fraction (*V*_f_) on the type of emulsion (water-in-oil –w/o– or oil-in-water –o/w–) in the presence of lecithin. Schematic presentation of the mayonnaise making process through phase inversion is given Figure 5. Phase inversion occurred by either increasing or decreasing *V*_f_, but a hysteresis under dynamic emulsification was observed; phase inversion (from w/o to o/w) occurs at high *V*_f_ (~0.5–0.6) when *V*_f_ was increased, while o/w to w/o inversion occurs at low *V*_f_ when *V*_f_ was decreased (~0.1–0.2). Dynamic emulsification seems to be a promising technique as it allows the incorporation of a high fraction of dispersed phase. This work could be completed by assessing emulsion stability with time, as this aspect was not taken into consideration by the authors; a study of interfacial behavior of lecithin could also be appropriate in order to determine, for example, the most favorable state of PL at the interface for phase inversion to occur.

In spite of the fact that phospholipids provide mostly o/w long-term stable emulsions, examples of w/o stable emulsions can also be found. Knoth *et al.* [89,90] have reported the preparation of water-in-oil emulsions using PC. Two point five percent PC dispersed in the oil phase was found to produce an emulsion which was stable over time but the dispersed phase flocculated, which was reversed by the application of shear. This flocculation was also noted to increase the viscosity of the emulsion. Addition of NaCl to the aqueous (dispersed) phase was reported to reduce the size of the dispersed phase droplets, but over time resulted in increased coalescence and sedimentation. This was attributed to increased adsorption of surface-active impurities and changes to the electrostatic behavior of lecithin.

Phospholipid–stabilized emulsions have mainly been investigated and produced for pharmaceutical applications. PL stabilized oil-in-water emulsions are widely used in intravenously administered medical treatments. These include parenteral emulsions for intravenous feeding and fat-soluble anesthetic compounds. These emulsions require a pH in the range of 7–8 and to be isotonic to allow intravenous use of large volumes. The first modern (1962) parenteral emulsion approved in Europe was Intralipid, which contains soybean oil, egg sourced phospholipids and glycerol, at a pH of 8. This emulsion was stabilized by the phospholipids and exhibits a dispersed (oil) phase droplet size below 1μm with lecithin used as the emulsifier [91]. Nowadays, the mean droplet size has been lowered to typically 200–500 nm [92]. Because formulations containing soybean lecithin exhibited undesirable reactions in some patients, the components causing this have been removed for modern use. Mixtures of safflower and soybean oil have also been used in other formulations more recently. Use of medium chain triglycerides as the oil phase has also occurred [92]. These changes to the oil phase formulation have, however, occurred for medical reasons only, not because of eventual alteration of physical properties of previously used emulsions. Compositions of 10%, 20% and 30% oil phase of Intralipid emulsions all report a fixed phospholipid concentration of 1.2%. This indicates a significant excess of phospholipids throughout the systems as this quantity suitably emulsifies 30% oil emulsions and must result in significant excess phospholipid when stabilizing a 10% oil emulsion. This excess has been shown to result in the presence of liposomes of a significantly smaller size (40 nm) than the emulsion droplets [93]. For medical reasons, there has been development of emulsions with lower phospholipid concentrations, the excess phospholipids in the 1.2% formulation being reduced [92].

pH effects on PL contained emulsion stability was assessed through studies by Han and co-workers aimed at controlling microbial activity [94,95]. Propofol, a lipid soluble anesthetic compound, is sold by many companies with differing formulations. For example, Diprivan is a 1% propofol preparation contained in a 20% oil-in-water emulsion stabilized by egg-derived phospholipid (1.2%). Han *et al.* [95] compared the stability of two different commercially available propofol emulsions presenting similar physical characteristics, but one exhibiting a lower pH due to the presence of antimicrobial additives. Lowering the pH reduces the zeta potential of the emulsion droplets due to changes in the ionization of the phospholipids. As a result, low pH emulsions showed poor stability during shaking and freeze–thaw tests, with extensive coalescence and phase separation, which was not observed at higher pH. Han and Washington [94] investigated the stability of Diprivan under a range of pH and ionic conditions and additives to the system to retard microbial growth. These conditional variations were tested with 16 h shaking of the emulsion following the changes. At a pH of 6.5 and 8 the system remained stable, while below 5 the system became unstable resulting in phase inversion. Emulsion disruptions were also observed when NaCl at concentrations higher than 25 mM or some antimicrobial agents were added to the emulsion.

Microbial activity was also studied for other drugs added to phospholipid–stabilized o/w emulsions for administration that include diazepam, etomidate and dexaneethasonepalmitate [92], clevidipine [82], or fat-soluble vitamins [96]. Emulsion stability against the addition of various components produced by human body (or living organisms more generally) has been tested. Addition of a wide variety of antimicrobials was undertaken by Sznitowska *et al.* [96], and these additions were observed to alter the emulsion stability on storage in some instances. Addition of divalent cations above a critical concentration to Intralipid emulsions has been reported to lead to the aggregation (creaming) but not coalescence of the dispersed phase [97], while glucose was found to be highly important in determining the stability of fat emulsions in total parenteral nutrition mixtures [98].

Temperature and pressure effects on the stability of phospholipid-based emulsions were investigated through the production of sterile emulsions for medical usage, which has been discussed in the work of many groups [94,99–102]. This has been achieved by the use of autoclave sterilization. The high temperature and pressure imposed by this process have not been observed to destabilize the phospholipid emulsions when used in some instances. As emulsions are thermodynamically unstable, the autoclaving process has been noted to accelerate degradation of the emulsions. Yu *et al.* [102] noted that sterilization at 121 °C for 15 min resulted in phase separation while 45 min at 105 °C and 30 min at 115 °C did not disrupt the emulsions. This acceleration of degradation can also be observed in the pH reported for autoclaved emulsions being significantly altered after the autoclaving process when a range of additives are present in the emulsion [96,103]. This reduction is attributed to the hydrolysis of phospholipids and triglycerides. An alternate method for the sterilization of o/w emulsions for medical usage is by filtration sterilization [96]. This does not require the high temperatures or pressures of autoclaving but does impart significant shear forces when passed through an aseptic 200 nm or 450 nm filter. This shear may destabilize the dispersed phase droplets resulting in aggregation or coalescence of the emulsion.

**Figure 6:**
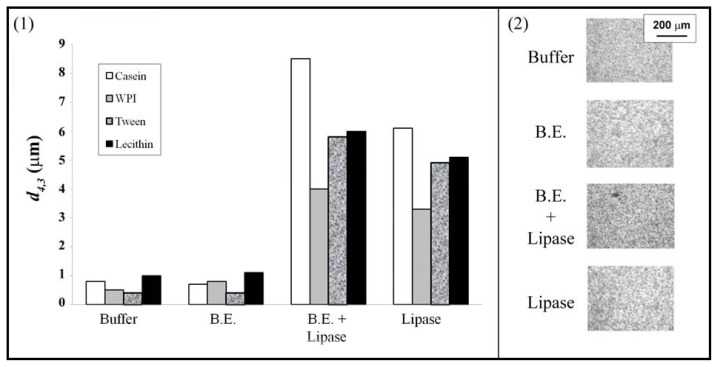
Influence of bile extract (B.E.) and lipase on (**1**) the mean particle diameter (*d*_4,3_) of 3 wt% corn oil-in-water emulsions (0.6 wt% sodium caseinate, WPI, Tween 20 and lecithin), and (**2**) the microstructure of 0.6% lecithin-stabilized emulsion (5 mM phosphate buffer, pH 7.0). Adapted with permission from Mun *et al.* [109], Copyright (2007) Elsevier.

Phospholipids have many applications in the food industry such as inhibitors of lipid oxidation [104] or food additives. They can also be used as emulsifiers, even though this topic is poorly documented. Lecithin, identified as the main stabilizer in mayonnaise in 1846, is the mostly used PL as a food emulsifier, if not the only one [105,106]. The wide variety of lecithins commercially available renders them suitable for stabilizing various types of emulsions. A few authors studied the response of PL stabilized emulsions to digestive conditions. Fillery-Travis and co-workers investigated the stability of olive oil-in-water emulsions in the presence of phosphatidylcholine and bile salt [107,108]. The authors demonstrated that bile salt interacted at the o/w interface with PC by altering the PL state at the interface; break-up of the LC state phospholipid layer resulted in a mixture PC/bile salt at the interface. This newly created interface enhanced emulsion stability as droplet flocculation was prevented, the presence of charged molecules (bile salt) at the interface promoting electrostatic repulsion. Bile salt also plays an antagonistic role in the interfacial enzymatic reaction. By breaking the PL structure, bile salt allows more lipase (enzyme) to bind at the interface; but bile salts in the continuous phase also bind lipase molecules, thus reducing their availability to adsorb at the interface. This work was pursued by Mun *et al.*, who investigated the effect of pancreatic lipase on lipid droplets for different interfacial compositions [109]. It was shown that, in the absence of bile, the lipase can access the interface for lecithin and proteins, but not for Tween 20 (Figure 6). The authors also confirmed that the introduction of bile extracts lead to a greater release of fatty acid for all the emulsifiers. In the presence of bile, the amount of fatty acid released was similar for emulsion prepared with Tween 20 and lecithin. Hypotheses explaining this behavior were formulated but no decisive conclusion could be made. As observed by Fillery-Travis *et al.* [107], PC emulsion stability is enhanced by bile extract. The authors, however, argued this was due to the displacement of most of (or all) PC from the interface; bile molecules play the role of high HLB value surfactant, similarly to Tween 20, and provide better stability against coalescence and creaming than lecithin. It was also noted that bile extracts disrupted Tween 20 or protein stabilized–emulsions.

Recently, the possibility of encapsulating pharmaceutical agents within food-grade emulsions was investigated by Li *et al.* [110] Tributyrin presents the peculiarity of being both triglyceride food additive and potentially an agent which prevents colon cancer. The stability of Tributyrin/corn oil-in-water was investigated for various emulsifiers and the effect of these emulsions on cancer cells was reported. Impact of emulsifiers on the cancer cell viability was first studied without tributyrin. Lecithin was shown to have the least impact on cell viability when used either in lecithin-in-water dispersion (no emulsion) or as emulsifier of corn oil-in-water emulsion. When tributyrin was incorporated in the emulsion, the authors showed that cancer cell viability was mainly driven by the presence of tributyrin.

For both pharmaceutical and food applications, attempts have been made to use mixtures of phospholipids and other emulsifiers to enhance emulsion stability. A number of co-surfactants have been reported as being used in conjunction with phospholipids to stabilise oil-in-water emulsions for pharmaceutical research. The most common of those is Pluoronic F68 (also called poloxamer 188), a polyoxyethylene-polyoxypropylene block copolymer [101]. Most commonly, the block copolymer is added to the water phase of the emulsion while the phospholipids are added to the oil phase, even though lecithins have also been reported as being prepared in the aqueous phase [96]. Yu *et al.* [102] have shown the improvements in emulsion stability with a total emulsifier concentration of 1.5% with varying concentrations of phospholipids and F68. The phospholipids act as an emulsifying agent while the F68 provides additional strength to the stabilized interface to resist processing conditions. Altering the total amount of emulsifier and co-emulsifier above or below 1.5% did not have a significant impact on the stability of the emulsion or the dispersed phase size. Zurowska-Pryczkowska *et al.* observed the doubling of the soybean oil-in-water emulsion droplet size when pilocarpine (active for glaucoma treatment) was encapsulated in the oil phase [103]. Addition of the co-surfactants Tween 80 or F68 returned the droplet size to that observed in systems without added pilocarpine. As opposed to Tween 80, emulsions with F68 were significantly less stable upon storage for 6 months; coalescence of dispersed phase droplets was measured in the presence of pilocarpine. Addition of F68 was also shown to improve the stability of emulsions containing physostigmine (an anticholinesterase). This was due to the formation of a mechanical barrier by the F68, as the physostigmine located at the interface tends to destabilize the emulsion [111].

Other co-emulsifiers have also been used. For example, polyethyleneglycol-660-hydroxystearate (PEG660) was used as a co-surfactant in the work of Jamaa and Müller [100]. Use of 0.75% of both lecithin and PEG660 resulted in a more stable emulsion system. The impacts of pH and electrolytes upon the lecithin were also shown to be reduced in a mixed system improving emulsion stability. Acetylated monoglycerides and Spans have also been reported as being used as co-surfactants in the preparation of emulsions for intravenous use [112]. Lecithin has been noted by Bylaite *et al.* to improve the stability and resistance to creaming in olive oil- and caraway essential oil-in-water emulsions stabilized by β-lactoglobulin [113]. These improvements are attributed to mixed surfactant layers with lecithin adsorbing to the interface without displacing protein, resulting in a reduction in the dispersed phase droplet size.

Donsì *et al.* studied the stability of the impact of freeze–thaw cycles on the stability of food grade oil-in-water nano-emulsions containing lecithin [114]. Lecithin-based emulsions were not stable to freeze–thaw cycle due to high droplet aggregation during freezing, which induces droplet coalescence. Nucleating protein and polyethylene glycerol, used as food additives, produced very different results. Emulsions in the presence of both lecithin and protein were even less stable to freeze–thaw cycles than when lecithin is used as sole emulsifier. On the contrary, emulsion stability was significantly enhanced by the addition of PEG; droplet size did not change even after 10 cycles.

Solid particles have also been associated to phospholipids to stabilize emulsions. Preparation of food grade o/w emulsions with soybean lecithin was demonstrated by Pichot *et al.* [115]. These emulsions, noted to be unstable within a few hours, were rendered stable by the addition of silica particles acting as “co-surfactant” Pickering particles. Lecithin was introduced from the oil phase while silica was dispersed in the aqueous phase. Emulsions were stabilized by silica particles, while lecithin adsorbed at the interface decreases the interfacial tension and delays re-coalescence of oil droplets during emulsification. Gouchi Eskandar *et al.* [116] have also reported the formation of o/w emulsions with soybean lecithin and silica. Lecithin at 0.1% resulted in less stable emulsions in comparison to those with added silica. The addition of silica from the oil phase was reported to result in more stable emulsions and a reduction in droplet size with increasing silica concentrations. Introduction of the silica from the aqueous phase exhibited no improvements to emulsion stability. With lecithin concentrations above 0.1%, the addition of silica did not increase emulsion stability.

### 3.2. Other Applications

**Figure 7:**
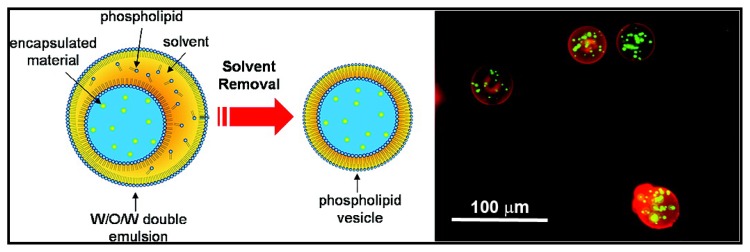
Schematic representation of the preparation of phospholipid vesicles using double emulsion (**left**) and fluorescent optical micrograph (**right**) of latex microspheres (green) encapsulated inside DPPC vesicles (red). Reprinted with permission from Shum *et al.* [120], Copyright (2008) American Chemical Society.

The stabilization of o/w interfaces by phospholipids is predominant in the literature. Nonetheless, PLs have also been used to form different structures such as particles or foams. Lecithin has been used as a surfactant to stabilize emulsions composed of pure triglycerides for the production of solid lipid nanoparticles [117,118]. The systems containing solid lipid nanoparticles exhibited less stability than an equivalent emulsion. These systems exhibited aggregation or gelling behavior upon cooling or under shear, which is not observed with the respective emulsions. Addition of co-surfactants was observed to reduce the aggregation of the solid nanoparticles and to prevent gelling. Crystallization of the triglycerides alters the dispersed phase shape (crystalline not spherical), increasing the surface area and disrupts the phospholipid surfactant coverage. These uncovered surfaces allow the aggregation and other behaviors of the solid lipid nanoparticles. The co-surfactants cover the “bare” lipid surface preventing aggregation. Sjöström and Bergenståhl also demonstrated that lecithin, mixed with sodium glycocholate (GCA), could produce and stabilize very small drug particles, as long as the ratio PC/GCA was kept between 9/1 and 2/1 [119] to avoid aggregation or undesired structures at the interface such as liposomes or mixed micelles. Optimum interfacial conditions for the production of stable particles are encountered when an extensive lamellar liquid-crystalline layer swells at the interface. The ability of PLs to stabilize both types of emulsion was used to develop stable water contained vesicles in aqueous media [120,121]. Preparation of a w/o/w system with the oil as a volatile solvent allows the evaporation of the solvent to create a thin phospholipid bilayer originating as a monolayer at both interfacial surfaces (Figure 7) [120]. The formation of gel beads coated by lecithin has been reported [122]. The mechanism to produce these beads includes (1) the formation of PL stabilized w/o emulsions above the gelling temperature of κ-carrageenan contained in the aqueous phase, (2) emulsion cooling to form the gel inside dispersed droplets, (3) the transfer of emulsion droplets through a PL monolayer formed at a planar o/w interface to produce a PL bilayer around the gel droplets. The final step ensures gel bead stability.

Most of the literature regarding foams stabilized by phospholipids is related to pulmonary surfactants and their impact in neonatal health. This will not be covered in this study, as this is a broad area and could be the topic of a full review. The literature of foams, as “dispersion of air bubbles in water”, stabilized by phospholipids is very scarce. Stabilization of water/air films by phospholipids and the impact a range of salts has upon these systems has been reported by Yamanaka *et al.* [123] Multivalent salts induce significant changes in the free energy of film formation. The a/w interfaces with dimyristoylphosphatidylcholine were seen to form “foam” films. The phospholipid is insoluble in water and so behaves as an insoluble particle with surfactant qualities. Films created with this system were observed to thin with increasing salt concentrations. Below the pH-induced isoelectric point, the films are not formed, with the reduced pH reducing the electrostatic interactions. Sarker *et al.* reported the improved stability of β-lactoglobulin aqueous solution foams with the addition of l-α-lysophosphatidylcholine [124]. This improvement occurred with lysolecithin concentrations from 0.5 to 7 times that of the lactoglobulin (1 mg/mL). Systems containing lysophosphatidylcholine concentrations above 15 mg/mg behaved similarly to a system without lactoglobulin. The mixed system was significantly more stable at lower phospholipid concentrations. Systems containing phospholipid only exhibit draining behavior like non-interacting surfactants.

## 4. Conclusion and Challenges

Understanding the role of phospholipids at fluid interfaces has generated a considerable amount of study for the last 50 years. This interest originated in the necessity to understand the structure and properties of biological membranes of which phospholipids are an important constituent, and has risen due to their amphiphilic character and natural sources of production. The large majority of the research on the subject is dedicated to the phospholipid behavior at the air/water interface. The main characteristic of PLs at the a/w interface is their ability to spread along the interface when this is stretched, due to their unique visco-elastic properties. PL spreading leads to changes in the state of the phospholipids at the interface from solid to gaseous, with various phases co-existing such as liquid expanded and liquid condensed. The well-understood behavior of phospholipids at the a/w interface resulted in a consequent number of papers related to the use of PLs as pulmonary surfactants. More generally, the pharmaceutical industry, more than any other industry, has developed phospholipid-based products. Nonetheless, the use of PLs for stabilizing foams has received only little interest, despite the increasing number of foam-based products commercialized, particularly by the food and cosmetic industries. This lack of studies is even more surprising considering the visco-elastic properties of PLs at the a/w interface and their changes of state as the interface is stretched.

The role of phospholipids at the o/w interface has also been studied, but to a much lesser extent than their role in a/w systems. This is mainly due to the increased difficulty of assessing the effect of PLs at the interface (surface pressure measurement or visualization) in the presence of oil; technological progress during the last couple of decades rendered possible the study of PLs at the o/w interface. Contrary to a/w systems, phospholipids tend to form multilayers at o/w interfaces. A key parameter in the understanding of the formation and structure of PL at the interface is the solubility of phospholipids in water and oil. Mechanisms of adsorption at the o/w interface are, however, not totally understood. This is due to the lack of studies and also to the fact that PC, within various forms, was the dominant PL studied. Completion of existing studies, replacing PC with other phospholipids, will foster a greater understanding of the interfacial phenomena.

Nonetheless, despite this limitation in understanding, phospholipids have been used as emulsifiers to stabilize o/w interfaces and form emulsions for the last 50 years. Due to their natural character, applications to pharmaceutical industry are numerous, and particularly related to parental emulsions. Applied researches, such as the production of sterile emulsions, also contributed to the understanding of PL behavior at the interface, at high temperatures or pressures. Phospholipids are also largely used in the food industry, as inhibitors of lipid oxidation or additives. Mixtures of PLs and co-surfactant or proteins to stabilize emulsions have received a lot of interest, and it would be very interesting to review this topic, but few studies have been published investigating the formation and stabilization of emulsions with PL as sole emulsifier. Phospholipids such as lecithin are natural food-grade surfactants and appear to have potential for expanded use in food due to their amphiphilic character and visco-elastic properties. With the growing interest for understanding food product microstructure, as well the necessity to develop new products, phospholipids could play a more important role as food emulsifiers in the near future. Despite the sparse but existing literature on PLs at the o/w interface, little use has been made of it to understand and explain emulsion stability. In particular, no attempt was made to relate the (in)stability of PL-stabilized emulsion when subjected to shear/stress to the ability of PLs to spread at the interface when expanded.

Even more surprising is that, to the best of our knowledge, there is no study that relates the formation of emulsion droplets in the presence of PLs, to the unique behavior of PLs at the interface. For example, one might wonder how the PL interfacial state affects the formation and stability of emulsion droplets formed by use of microfluidics instruments or membrane emulsification devices.

## Abbreviations

α-DML: α-Dimyristoyllecithin
α-DPL: α-Dipalmitoyllecithin
β-DPL: β-Dipalmitoyllecithin
DHPC: Dihexadecylphosphatidylcholine
DLPC: l-α-dilauroylphosphatidylcholine
DMPA: Dimyristoylphosphatidic acid
DMPE: l-α-Dimyristoyl phosphatidylethanolamine
DOPC: 1,2-dioleoyl-3-*sn*-phosphatidylcholine
DSPC: distearoylphosphatidylcholine
DPPC: Dipalmitoyl phosphatidylcholine
LC: Liquid-Condensed phase
LE: Liquid-Expanded phase
PA: Phosphatidic Acid
PC: Phosphatidylcholine
PE: Phosphatidylethanolamine
PG: Phosphatidylglyerol
PI: Phosphatidylinositol
PL: Phospholipid
PS: Phosphatidylserine
